# Supplementary figures and images for: Laminarin protects against hydrogen peroxide-induced oxidative damage in MRC-5 cells possibly via regulating NRF2
Source: PeerJ. 2017 Jul 31;5:e3642. doi: 10.7717/peerj.3642 (PMC5541921; doi:10.7717/peerj.3642)

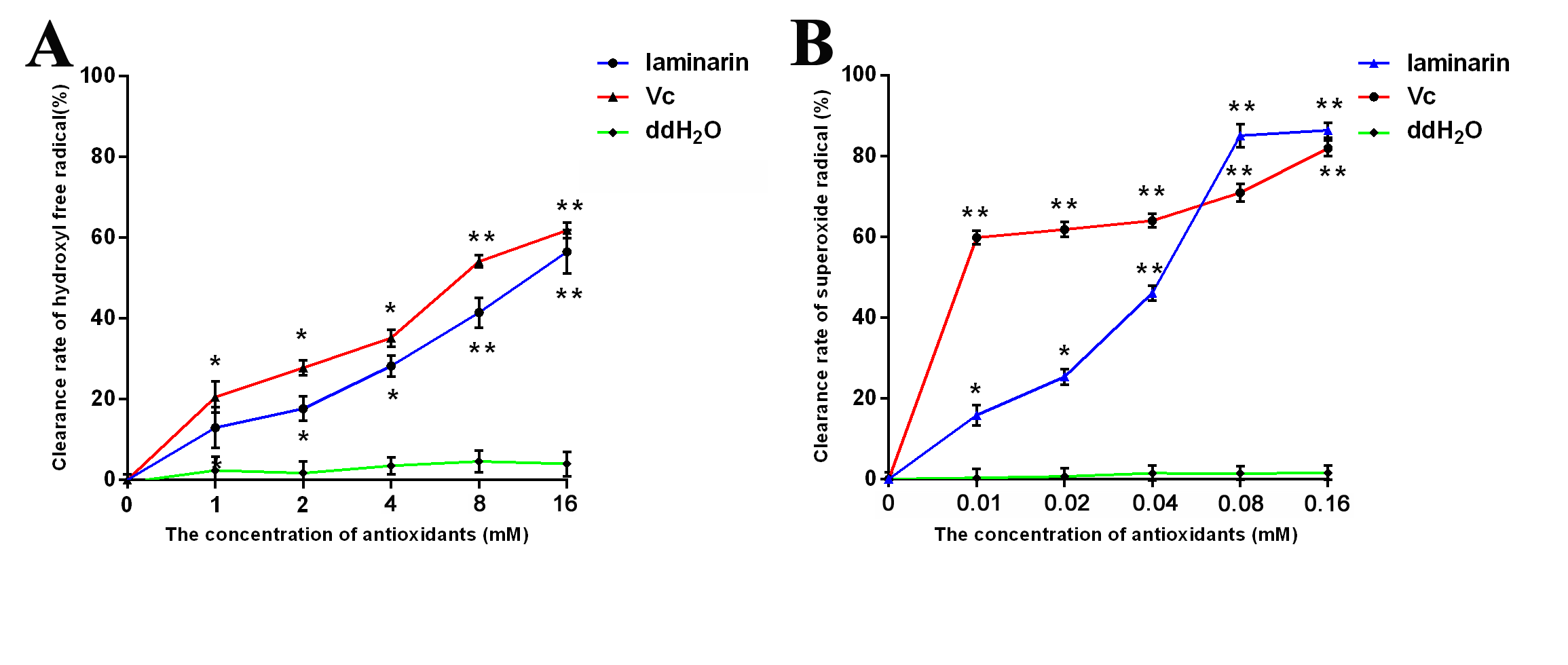

Supplement: Figure S1 — (A) The clearance rate of hydroxyl free radical. (B) The clearance rate of superoxide radical. Data are shown as mean ± SD (n = 6). * P < 0.05, ** P < 0.01 compared with control group. [file peerj-05-3642-s001.png]
